# *Arthrobacter woluwensis* Bacteremia: A Clinical and Genomic Report

**DOI:** 10.3390/pathogens10040443

**Published:** 2021-04-08

**Authors:** Shu-Yuan Li, Chin-Chuan Kao, Yu-Cheng Hu, Chung-Hsu Lai, Yi-Ping Jiang, Yan-Chiao Mao, Yao-Ting Huang, Po-Yu Liu

**Affiliations:** 1 Department of Infectious Diseases, Taichung Veterans General Hospital, Taichung 40705, Taiwan; saaoc2001@gmail.com (S.-Y.L.); fiorano790502@gmail.com (Y.-C.H.); 2 Division of Infectious Disease, Department of Internal Medicine, Tungs’ Taichung Metroharbor Hospital, Taichung 433402, Taiwan; cylindroma@hotmail.com.tw; 3 Division of Infectious Diseases, Department of Internal Medicine, E-Da Hospital, Kaohsiung 840, Taiwan; laich6363@yahoo.com.tw; 4 School of Medicine, College of Medicine, I-Shou University, Kaohsiung 840, Taiwan; 5 Department of Computer Science and Information Engineering, National Chung Cheng University, Chiayi 62102, Taiwan; apple80177@gmail.com; 6 Division of Clinical Toxicology, Department of Emergency Medicine, Taichung Veterans General Hospital, Taichung 40705, Taiwan; doc1385e@gmail.com; 7 School of Medicine, National Defense Medical Center, Taipei 11490, Taiwan; 8 Rong Hsing Research Center for Translational Medicine, National Chung Hsing University, Taichung 402, Taiwan; 9 Ph.D. Program in Translational Medicine, National Chung Hsing University, Taichung 402, Taiwan

**Keywords:** *Arthrobacter woluwensis*, bacteremia, *ureC*

## Abstract

*Arthrobacter woluwensis* is a Gram-positive, aerobic *Actinobacteria* that is widely distributed in the environment worldwide. Little is known about *A. woluwensis* infection and it is commonly mis-identified by culturing with commercial kits. To date, only six cases of bacteremia caused by *A. woluwensis* have been reported in the literature. Herein, we report a case of *Arthrobacter woluwensis* bacteremia in an immunocompromised host. In this case report, the results of antimicrobial susceptibility testing showed that this clinical isolate of *A. woluwensis* is sensitive to vancomycin, teicoplanin, but resistant to penicillin, cephalosporin and ciprofloxacin. Additionally, whole genome sequencing analysis identified common subunits of the urease system.

## 1. Introduction

*Arthrobacter woluwensis* is a Gram-positive, aerobic *Actinobacteria* that is widely distributed in the environment, mainly in soil, and it sometimes represents the majority of single bacterial groups in aerobic plate counts of soil specimens [1]. *Arthrobacter* strains have relatively low pathogenic potential but they can be pathogenic to immunocompromised hosts. *Arthrobacter* spp. was first considered as a *Corynebacterium* in 1896 when the species *Corynebacterium* was first proposed, but it was later assessed to be a distinct species [2]. Considering its rarity and similarity to other corynebacteriae, it is commonly mis-identified in culturing by commercial kits, thus, proper identification often requires assistance from molecular biological methods, such as chemotaxonomic methods or ribosomal RNA/DNA sequencing. To the best of our knowledge, only six cases of bacteremia related to *Arthrobacter woluwensis* have been reported to date [1,3,4,5,6,7].

Here, we present a case of a newly diagnosed gastric cancer patient who suffered from *Arthrobacter woluwensis* bacteremia during their hospital admission.

### 1.1. Specimen Collection and Antibiotic Susceptibility Testing

A 93-year-old man received total gastrectomy, D1 lymph node dissection, and Roux-en-Y anastomosis due to adenocarcinoma of the stomach and developed a postoperative wound infection. Blood culture from peripheral blood yielded unidentified Gram-positive bacillus. The strain was designated as QTS. Antimicrobial susceptibility testing of the isolate was performed with VITEK2 (bioMérieux) and the strain was susceptible to penicillin, vancomycin, trimethoprim/sulfamethoxazole (TMP/SMX), and resistant to ciprofloxacin, clindamycin, and gentamicin. E test MIC values of penicillin, vancomycin, amikacin and daptomycin were 0.75 µg/mL, 1.0 µg/mL, 2 µg/mL and 3 µg/mL. 

### 1.2. Genome Sequencing, Assembly, Annotation, and Phylogenetic Analysis

One colony of trypticase soy agar with 5% sheep blood was transferred into 5 mL of Müller–Hinton medium and incubated overnight at 37 °C. DNA isolation was done according to the manufacturer’s specifications with the QIAGEN Genomic-tip 100/G100 kit and the Genomic DNA Buffer (Gentra Bio, Paisley, UK), measuring the DNA concentration with a Qubit 2.0 fluorometer (Life Technologies, Carlsbad, US). The QTS whole genome was sequenced by Oxford Nanopore GridION using R9.4 flow cell at 122x coverage. The raw signals were basecalled by Guppy 3.4 into long reads. The adaptors that remained in the long reads were trimmed by Porechop. These clean reads were assembled by Flye v. 2.6 into a circular chromosome of 3.68 Mbp. The sequencing errors left on the genome were polished by four runs of Racon, one run of Medaka, and finally, one run of Homopolish. The resulting genome nearly reached 100% CheckM completeness. The protein-coding genes in the QTS genome were annotated via the NCBI Prokaryotic Genome Annotation Pipeline (PGAP). Virulence factors in the genome were identified by DIAMOND alignment against the virulence factor database (VFDB). Whole-genome average nucleotide identity (ANI) was computed by OrthoANI. The phylogeny of QTS and other related genomes was reconstructed by MEGA X. The circular comparative genome map was plotted by Circos.

## 2. Result

**Figure 1:**
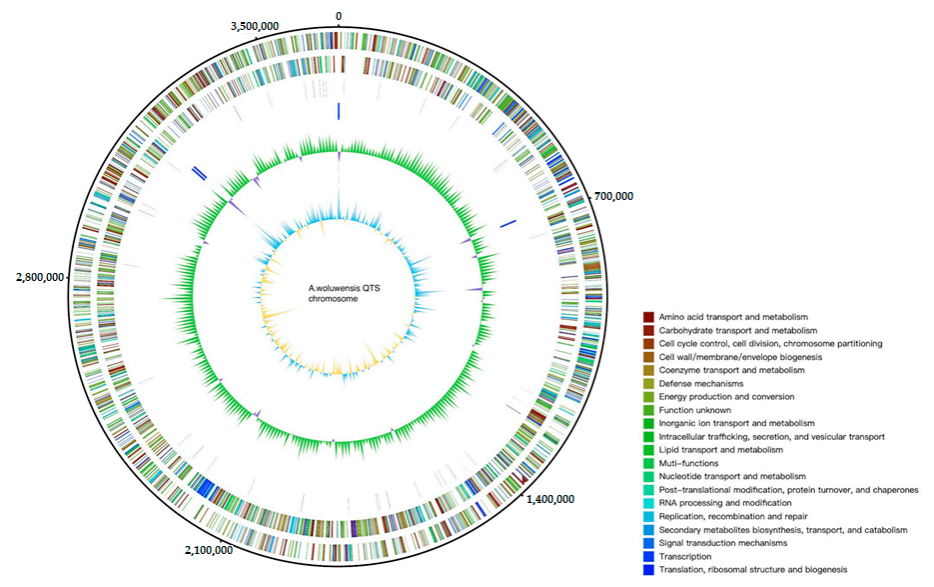
A graphical circular map of *Arthrobacter woluwensis* QTS. Graphical depiction from outside to the center: DNA coordinate, protein-coding genes on forward strand and reverse strand (colored by COG categories), tRNA gene, rRNA gene, GC content and GC skew.

**Figure 2:**
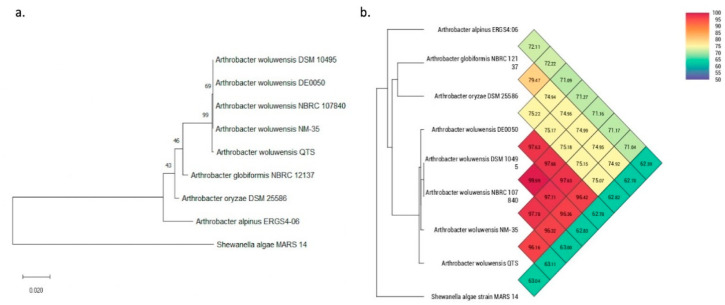
Phylogenetic analysis using 16S rRNA and ANI. (**a**) Phylogeny reconstruction using 16S rRNA of QTS and seven closely-related species under the *Arthrobacter* genus. *Shewanella algae* MARS14 is used as an outgroup. The bootstrap values and branch length are shown on the nodes and edge, respectively. (**b**) Phylogenetic cluster using pairwise ANI between the eight *Arthrobacter* species and the outgroup genomes. The heat map contains pairwise ANI of any two genomes. QTS shares >96% ANI with four *A woluwensis* genomes.

**Figure 3:**
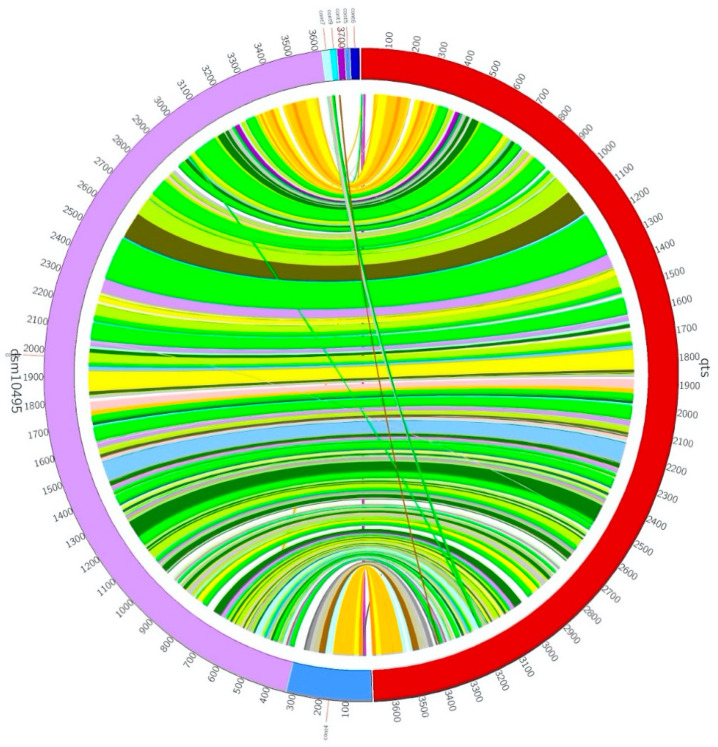
Circos plot of genomes of *A.woluwensis* QTS and *A.woluwensis* DSM 10495.

The information regarding gene sequencing and assembly is summarized in Table 1. The genomic contents of the QTS genome are illustrated in Figure 1. The phylogenetic tree based on the 16S rRNA sequence and ANI was constructed to show the phylogenetic position of *A. woluwensis* QTS (Figure 2). *A. woluwensis* QTS is closely related to other *A. woluwensis* strains in terms of nucleotide sequences, sharing an ANI > 96%. As shown in Figure 3, the alignment revealed an obvious syntenic relationship between strains QTS and DSM 10495. Candidate virulence genes are presented in the Supplementary Materials, Table S1, including urease operon. Common subunits of the urease system were identified, including *ureA*, *ureC*, *ureD*, *ureE*, *ureF* and *ureG* [8].

## 3. Discussion

Pubmed was used to search the literature published before March 2021 with the keywords: *Arthrobacter woluwensis*, *Arthrobacter*, and bacteremia. The characteristics of patients, diagnosis, treatment and outcome are summarized in Table 2.

Four of six patients in the literature review demonstrated features of poor immunity, including AIDS, terminal colon cancer, extreme old age and multiple myeloma under chemotherapy treatment. In 3 of 5 patients, a central venous catheter was in place when bacteremia occurred. Case 3 and 6 were intravenous drug users and vulnerable to bloodstream infection. 

The antimicrobial susceptibility is summarized in Table 3. Most isolates showed sensitivity to vancomycin, teicoplanin, tetracycline and showed resistance to penicillin, cephalosporin, gentamicin and ciprofloxacin. 

In the literature review, patients with immunocompromised status and central venous catheter insertion were vulnerable to *A. woluwensis* bacteremia. *Arthrobacter* strains have been isolated from aqueous/vitreous fluid [9], placenta [10], inguinal lymph node [11], implantable cardioverter defibrillator cultures [12], wound swab [13], urine, cervix, vaginal swab, neck abscess [14], and peritoneal dialysis fluid [15]. *A. cumminisii* and *A. oxydans* were the most common species found in human clinical specimens [14]. Despite being found in various organs in the human body, they rarely cause infections, indicating the low pathogenicity of *Arthrobacter* spp. 

In the present study, commercial kits failed to identify *Arthrobacter* spp. Further phenotypic features and genotyping methods may help with more precise identification. Due to the lack of precision of the identification methodologies, the true incidence of infection caused by *Arthrobacter* spp. may be underestimated. 

The patients mentioned in the literature were treated successfully with ampicillin, vancomycin, linezolid, and teicoplanin. However, MIC results were variable and antibiotic resistance to β-lactam and ciprofloxacin was found. Relatively high vancomycin MIC was observed in some isolates. Empirical therapy for these cases can be challenging before MIC results are available. In most of the patients, a central venous catheter was in place when *A. woluwensis* bacteremia occurred. Multidimensional central-line bundle care and catheter removal may be an essential measure in the prevention and treatment of *A. woluwensis*-related infection. It was reported that central line-associated bloodstream infection was significantly reduced by 12.2% after care bundle implementation in intensive care units in Taiwan [16]. 

**Figure 4:**
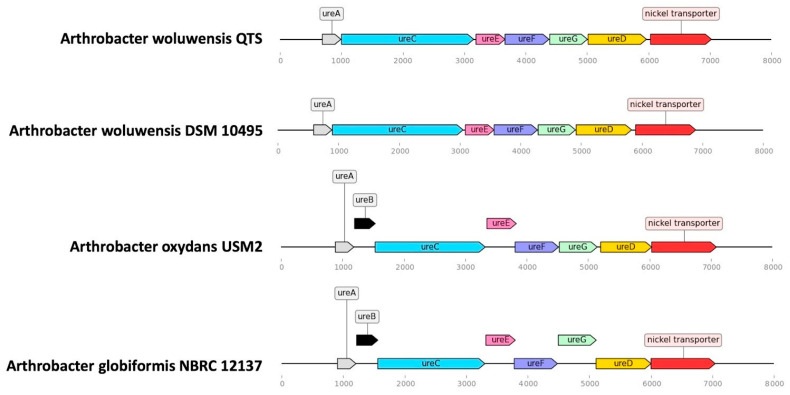
*Arthrobacter woluwensis* QTS and several *Arthrobacter* spp. contained a similar gene structure associated with urea hydrolysis.

Urea hydrolysis has been observed in *Arthrobacter woluwensis* isolates in previous reports [1,3,4,6] and in some other *Arthrobacter* spp. [1,17]. Previous studies have reported that bacterial urease and associated proteins were encoded by *ureA*, *ureB*, *ureC*, *ureD*, *ureE*, *ureF* and *ureG*, as shown in Figure 4 [8]. *UreC* gene was found in the isolate of this report. Friedrich et al. reported *ureC* gene was more frequent among Shiga toxin-producing *E. coli* (STEC). The clinical STEC isolates contained *ureC* but seldom expressed urease activity [18]. Urease is an important factor for *Helicobacter pylori* colonization in human gastric mucosa [19]. Hence, *ureC* gene could be used for *H. pylori* detection and was a potential target for vaccine production and therapeutic antibody [20,21,22]. The association between urease genes and the virulence of *Arthrobacter* spp. is still unclear. 

## 4. Conclusions

Here, we report a case of *Arthrobacter woluwensis* bacteremia in an immunocompromised host treated successfully with ampicillin and catheter removal in Taiwan. Whole genome sequencing identified common subunits of the urease system. 

## Figures and Tables

**Table 1:** Sequencing and assembly.

Strains	Coverage	Genome Size	Genes	Sequencing Technology	Assembly Method	Country	Host	Isolation Source	DNA	Accession Number
QTS	122.0x	3680971	3369	Oxford Nanopore GridION	Flye v. 2.6	Taiwan	Homo species	Blood	Circular	CP049819

**Table 2:** Reported cases of *Arthrobacter woluwensis* bacteremia.

Case No.	Age/Sex	Underlying Condition	Diagnosis	Specimen	Treatment	Risk Factor	Outcome
1 [1]	33F	HIV infection, stage C-3	Bacteremia	Blood	2 weeks ampicillin	Port-A catheter	Survival
2 [6]	56M	Metastatic colon cancer	Bacteremia	Blood	Vancomycin, catheter removal	Subclavian catheter	Died ^1^
3 [3]	39M	IVDU, TB	Mitral valve endocarditis	Blood	6 weeks teicoplanin	IVDU	Survival
4 [4]	91F	Ischemic stroke	Bacteremia	Blood	10 days linezolid	Hospital acquired infection	Survival
5 [5]	76F	Multiple myeloma, HTN, DM	Bacteremia	Blood	19 days teicoplanin, catheter removal	Chemoport	Survival
6 [7]	52	Hepatitis C	Mitral and aortic valve endocarditis	Intraoperative samples and blood	Operation ^2^, Teicoplanin, TMP/SMX, linezolid ^3^	IVDU	Survival
Present case	93M	CAD, HTN, prostate cancer, newly diagnosised gastric cancer	Bacteremia	Blood	2 weeks ampicillin	CVC, post-OP wound	Survival

^1^ Died due to underlying malignancy. ^2^ Mitral valve replacement with a biological prosthetic valve and an aortic vegetectomy. ^3^ Teicoplanin + TMP/SMX after operation, switch to linezolid and TMP/SMX before discharge, total 4 weeks course of treatment following operation. HIV: human immunodeficiency virus; IVDU: Intravenous drug user; TB: tuberculosis; HTN: hypertension; DM: diabetes mellitus; TMP/SMX: Trimethoprim/sulfamethoxazole; CAD: coronary artery disease; CVC: central venous catheter.

**Table 3:** Antimicrobial susceptibility profile of *Arthrobacter woluwensis* in reported cases.

Case	AMC	AM	CRO	CXM	CE	CIP	CLI	ERY	GM	IPM	PE	RIF	TEC	TET	VA	DAP	LIN
1 [1]	R ^#^	R	R	R	R	R	R	R	R	R	R(4 *)	R	S	S	S(2)		
2 [6]											R(4)				S(1.5)		
3 [3]		R	R	R	R	R	I		I		R(4)	S	S	S	S(2)		
4 [4]						S	S	S			S	I	S		S		S
5 [5]								I	R		I(1)				S(2)		
Present case						R	R		R		I(0.75)				S(1)	R	

AMC, amoxicillin-clavulanic acid; AM, ampicillin; CRO, ceftriaxone; CXM, cefuroxime; CE, cefalothin; CIP, ciprofloxacin; CLI, clindamycin; ERY, erythromycin; GM, gentamicin; IPM, imipenem; PE, penicillin G; RIF, rifampin; TEC, teicoplanin; TET, tetracycline; VA, vancomycin; DAP, daptomycin; LIN, linezolid. ^#^ According to CLSI breakpoints. * (): MIC result.

## Data Availability

This Whole Genome Shotgun project has been deposited at GenBank under the accession CP049819.

## Supplementary Materials

The following are available online at https://www.mdpi.com/article/10.3390/pathogens10040443/s1, Table S1: Candidate virulence genes.
